# Impact of electroacupuncture on quality of life for patients with Relapsing-Remitting Multiple Sclerosis under treatment with immunomodulators: A randomized study

**DOI:** 10.1186/1472-6882-12-209

**Published:** 2012-11-05

**Authors:** Juan G Quispe-Cabanillas, Alfredo Damasceno, Felipe von Glehn, Carlos O Brandão, Benito P Damasceno, Wanderley D Silveira, Leonilda MB Santos

**Affiliations:** 1 Neuroimmunology Unit, Department of Genetics, Evolution and Bioagents, University of Campinas (UNICAMP), Campinas, SP, Brazil; 2 Department of Clinical Medicine, University of Campinas (UNICAMP), Campinas, SP, Brazil; 3 Department of Genetics, Evolution and Bioagents, University of Campinas (UNICAMP), Campinas, SP, Brazil; 4 Department of Neurology, University of Campinas(UNICAMP), Campinas, SP, Brazil

**Keywords:** Multiple sclerosis, Electroacupuncture, Pain, Depression, Chinese traditional medicine, Symptoms, Quality of life, Randomised control trial, Expanded disability status scale

## Abstract

**Background:**

Multiple sclerosis (MS) is a complex autoimmune disease mediated by an immune response to central nervous system antigens. Modern immunomodulatory therapies, however, do not ameliorate many of the symptoms, such as pain and depression. Patients thus seek alternative treatments, such as acupuncture, although the benefits of such treatments have not been objectively evaluated. The present study was thus designed to evaluate the effect of the use of acupuncture in the alleviation of the symptoms of patients with MS.

**Methods:**

Thirty-one patients with Relapsing-Remitting Multiple Sclerosis undergoing treatment with immunomodulators were randomly distributed into sex-stratified experimental and placebo groups in a patient- and evaluator-blind design; they received either true or sham electroacupuncture during regular visits to the doctor in the university hospital outpatient clinic. Standardized questionnaires were used to evaluate the effect of electroacupuncture on the quality of life of these patients. Initial and follow-up assessment included the evaluation of clinical status (Expanded Disability Status Scale), pain (Visual Analogue Scale) and quality of life (Functional Assessment of multiple Sclerosis) to ascertain the impact of electroacupuncture on the quality of life of these patients.

**Results:**

Electroacupuncture improved various aspects of quality of life, including a reduction in pain and depression. The self-report scales were more sensitive to improvement than was the more objective clinical measure.

**Conclusion:**

This paper provides evidence that electroacupuncture can significantly improve the quality of life of such patients. The results suggest that the routine use of a self-report scale evaluating quality of life should be included in regular clinical evaluations in order to detect changes more rapidly.

**Trial Registration:**

RBR-58yq52

## Background

Multiple sclerosis is a complex autoimmune disease mediated by the individual immune response against central nervous system (CNS) antigens. Its causes are unknown, although, both genetic and environmental components play important roles and interact to produce susceptibility to the disease and influence its course [1]. For the relapsing-remitting type of multiple sclerosis (RRMS), various immunomodulatory therapies have been developed, with an effectiveness of some 35%, and these have had a significant impact on the natural history of the disease [2-4]. However, although these agents do reduce the relapse rate, they do not ameliorate many of the symptoms and lead to undesirable side effects [5]. Patients may still complain of diverse symptoms, such as difficulty in walking, incontinence, impaired sexual function, and pain, all of which lead to a poor quality of life (QOL) [6], and many seek alternative treatments such as acupuncture. Acupuncture, which is an important component of Traditional Chinese Medicine (TCM), involves the insertion of metallic needles into specific points, stimulating them either manually or electrically (eletroacupuncture) [7]. Its increase in popularity during recent years has not gone unnoticed by the medical community with clinical trials using acupuncture for the alleviation of pain and stroke rehabilitation [8-12]. Nevertheless, few reports address the benefits of this treatment on the quality of the life of RRMS patients [13]. Our aim was thus to study the effect of electroacupuncture on the QOL of patients with RRMS undergoing treatment with immunomodulators, using standardized questionnaires to evaluate these effects.

## Method

### Subjects

**Figure 1:**
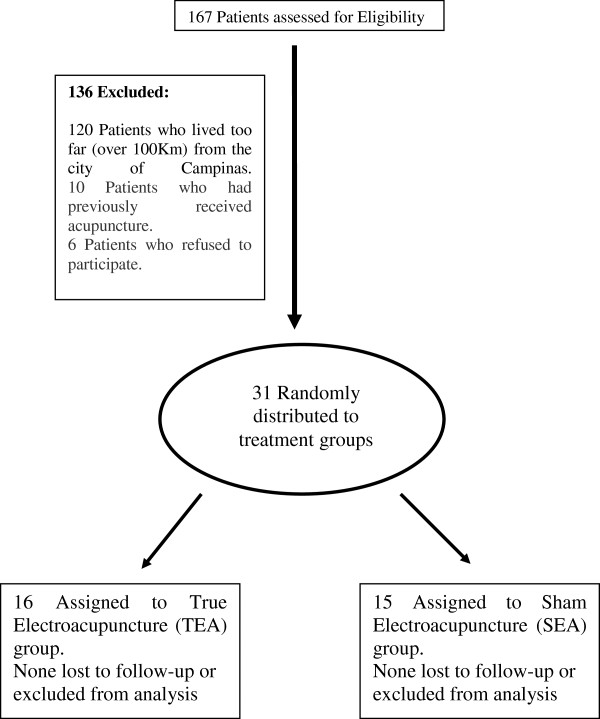
Flow diagram outlining participant selection.

The study was carried out in the MS outpatient clinic of the University of Campinas Hospital (UNICAMP) in Campinas in the State of São Paulo in Brazil between 2008 and 2010. A total of 167 patients with a confirmed diagnosis of RRMS according to the revised 2010 McDonald criteria and under treatment with immunomodulatory drugs (interferon-beta and glatiramer acetate) were identified [14,15]. The sample selected consisted of all 31 living close enough to appear for weekly appointments and who had not previously received acupuncture treatment (Figure 1); these subjects were distributed into true acupuncture and placebo (sham acupuncture) groups. All patients provided informed consent and agreed to receive one of two interventions, either true electroacupuncture (TEA) or sham electroacupuncture (SEA). They were informed that it was possible that this procedure might or might not have any effect on their condition. The study was approved by the University of Campinas Committee for Ethical Research CAAE - 0775.0.146.000-08 and The Brazilian Clinical Trials Registry (RBR-58yq52, http://ecgovbr2.bvsalud.org/).

### Randomization and blinding

Simple randomization to allocate patients to equal parallel groups receiving TEA or SEA treatments was based on a table of random numbers, with the groups stratified by sex. The study involved a patient- and evaluator-blind design. Only the practitioner, an experienced acupuncturist with more than ten years of experience and accredited by the Brazilian National Council for Biomedicine in the area of acupuncture, was aware of the intervention to be used. Treatment was applied following individual schedules so that none of the patients had any contact with the others in the study. Moreover, all patients agreed not to have contact with other participants and to avoid talking about their treatment. Initial assessment included the application of three previously validated instruments to evaluate 1) clinical status (Expanded Disability Status Scale), 2) pain (Visual Analogue Scale) and 3) quality of life (Functional Assessment of multiple Sclerosis).

### Intervention

All patients were receiving conventional treatment for RRMS, which involves a self-administered daily injection of interferon beta 1-a or interferon beta 1-b, and this treatment was continued throughout the present study [2-4]. Acupuncture was initiated after the determination of the two groups. The protocol consisted of 30 minutes of electroacupuncture (either true or sham) once a week for six consecutive months.

A single treatment protocol was developed for all patients on the basis of acupuncture points reported to stimulate the immune system [16-21]. Disposable stainless steel needles (diameter 0.20 mm x length 30 mm; Dongbang, Republic of Korea) were used. For the experimental group, the needles were inserted bilaterally at the points Zusanli (ST36), Sanyinjiao (SP6), Hegu (LI4) and Quchi (LI11), with a ninth needle inserted at Yintang (EX-HN3); the location of the points, angle of insertion and depth of insertion were based on the references in a classic text of Chinese medicine [22]. The “de qi” (manifestation of sensation in response to the presences of the needle) was sought for each point. In the SEA group, needle insertion was more superficial (less than 0.2 cm) and one centimeter to the side of the critical points used for the TEA group, with care taken to avoid areas corresponding to any of the 14 meridians of TCM. After the needles were inserted, they were connected to the terminals of a six-channel KWD 808 serial impulse electrostimulator (Changzhou, China) set to run on alternating current and produce an electrical stimulus of 4 Hz in discontinuous waves, with a pulse width of 0.5 ms. The stimulation was applied to each of the needles (except for that in Yintang) for the TEA group, while for the SEA group, no actual electrical stimulation was given.

### Outcome measures

Clinical status was assessed prior to the treatment, as well as after six months, using the Expanded Disability Status Scale (EDSS), which is an instrument used to evaluate neurological impairment on a scale of 0 (normal capacity) to 10 (death) [23]. Quality of life was also assessed prior to the initiation of treatment, as well as after three and six months; it was measured by the Functional Assessment of Multiple Sclerosis (FAMS) instrument, validated for Portuguese [24,25]. Briefly, this is a disease-specific 44-item self-report questionnaire investigating patient perception of the quality of their lives, using a 5-point Likert-type scale ranging from “not at all” to “very much”, which yields a score between 0 (for not at all) and 4 (for very much) for each of the items. The six sub-scales assess mobility, symptoms, emotional well being/depression, general contentment, thinking/fatigue, and family/social well being; moreover, patient observations of perceived changes in their lives with treatment were also noted. Pain was evaluated using the 10-point Visual Analogue Scale (VAS), applied just before each application of the FAMS. Miscellaneous improvements were occasionally mentioned by patients, and these were also noted.

### Statistical analysis

Data were analyzed using SAS System for Windows software, version 9.2 (SAS Institute, Cary, NC, USA), with a Fisher′s exact test used to determine differences between proportions. The Mann–Whitney test was used for between-group comparisons of the parameters evaluated. An analysis of variance (ANOVA) test with repeated measures was used for between-group comparison of the ranked parameters, as well as to ascertain differences due to length of treatment. The statistical tests were two-tailed, with level of significance set at 5%.

## Results

Figure 1 provides a flow diagram of the selection and treatment of the two groups, with sixteen in the TEA group and fifteen in the SEA. The groups were similar with respect to age, gender, and duration of the disease (Table 1).

**Table 1:** Participant characteristics

**Treatment Group**	**Age**			**Gender**		
	**#**	(years*)	**SD**	**F/M**	Years with MS*	**SD**
SEA^1^	15	40.1	(9.1)	13/2	9.3	(7.0)
TEA^2^	16	36.0	(11.5)	14/2	7.6	(6.0)

*Mean, ^1^sham electroacupuncture (SEA), ^2^true electroacupuncture (TEA).

### Clinical status

**Figure 2:**
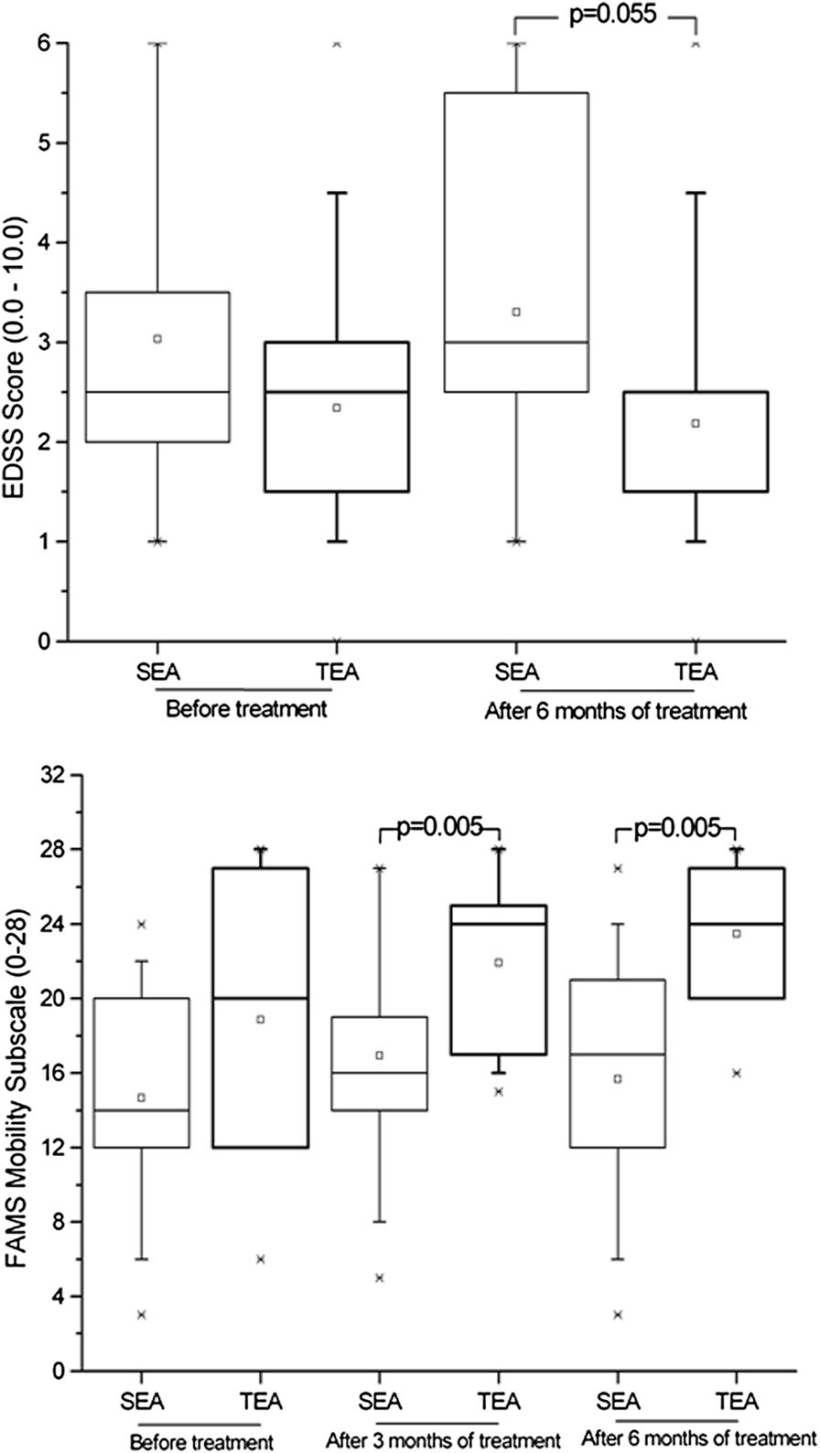
Assessment of neurological involvement of patients, including mobility, based on results of Expanded Disability Status Scale (EDSS) administered before treatment and after six months of weekly electroacupuncture (or sham electroacupuncture) sessions and of self-assessed mobility as revealed by the mobility subscale of the Functional Assessment of Multiple Sclerosis questionnaire (FAMS).

The clinical evaluations based on the EDSS were similar for the two groups at the beginning of the study (initial means of 3.0 and 2.3 for the SEA and TEA groups, respectively (p=0.3). After 6 months of treatment, however, the group receiving the SEA had shown somewhat more deterioration, although the difference between the two groups only approached significance (3.3 vs 2.2; p = 0.055). Nevertheless, when including the effect of duration of treatment using the ANOVA test, there was a significant interaction effect, with the patients receiving the sham treatment receiving higher (worse) scores than those receiving true electroacupuncture (P_interaction effect_ = 0.0338; Figure 2).

### Quality of life

**Figure 3:**
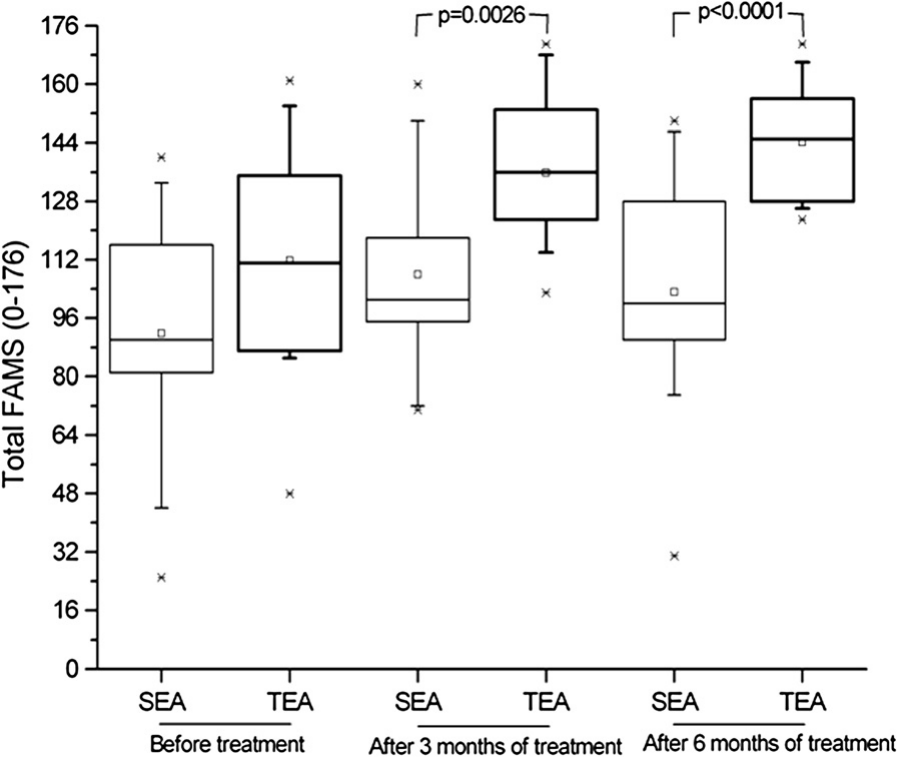
Overall Results of Functional Assessment of Multiple Sclerosis questionnaire (FAMS) administered prior to treatment, as well as after three and six months of weekly electroacupuncture (or sham electroacupuncture) sessions.

**Figure 4:**
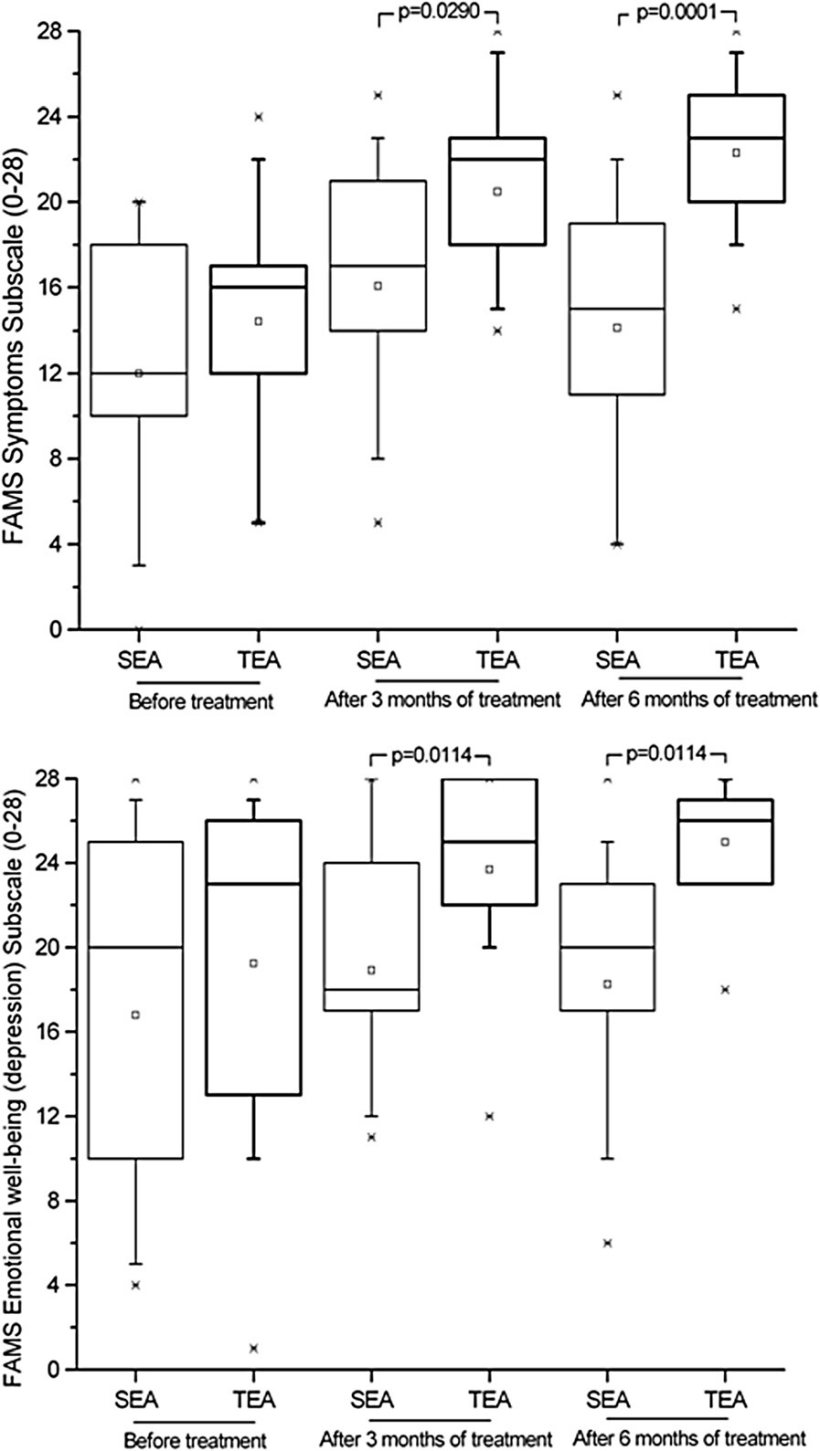
Assessment of Quality of Life based on Symptoms and Emotional well-being (depression) subscales of the Functional Assessment of Multiple Sclerosis questionnaire (FAMS), administered prior to treatment, as well as after three and six months of weekly electroacupuncture (or sham electroacupuncture) sessions.

**Figure 5:**
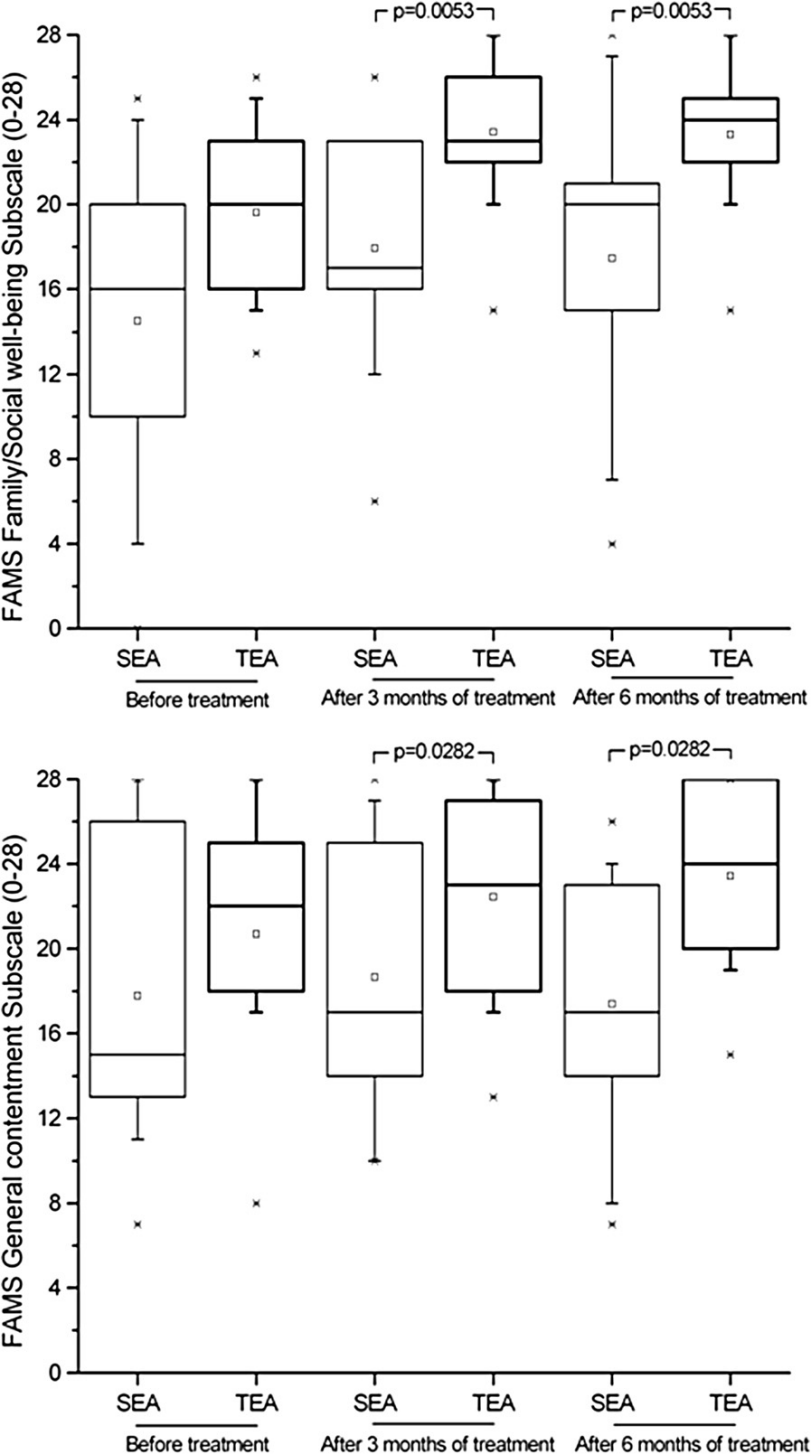
Assessment of Quality of Life based on Family/Social well-being, and General Contentment subscales of Functional Assessment of Multiple Sclerosis questionnaire (FAMS), administered prior to treatment, as well as after three and six months of weekly electroacupuncture (or sham electroacupuncture) sessions.

Although initial QOL scores for all subscales were similar for the two groups (with p-values ranging from 0.0985 to 0.5333), after treatment there was a significant difference between the groups (p-values of 0.0026 and <0.0001 after three and six months, respectively). Only the thinking/fatigue subscale showed no significant difference between groups after treatment (p = 0.073 for both time periods). These improvements are shown in Figures 2, 3, 4 and 5. The SEA group did show a transitional improvement on the symptoms subscale after three months of treatment (p < 0.01), but this did not persist to the end of treatment (p > 0.10).

### Pain

**Figure 6:**
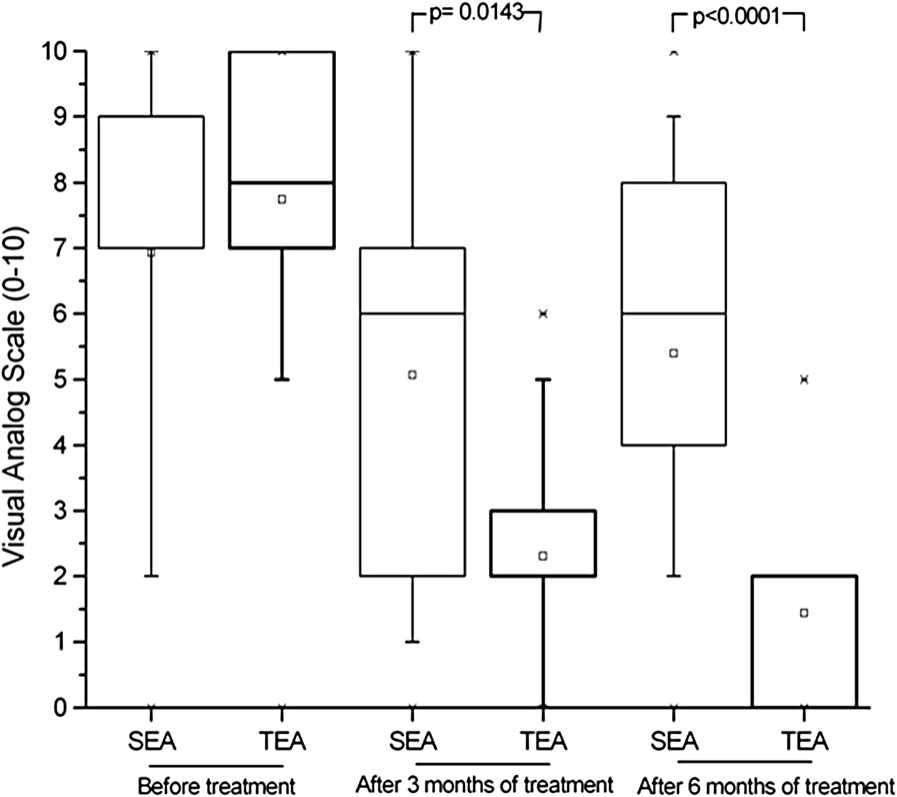
Assessment of pain based on results of Visual Analogue Scale (VAS) administered prior to treatment, as well as after three and six months of weekly electroacupuncture (or sham electroacupuncture) sessions.

The presence of pain, evaluated by the VAS, was similar for the two groups prior to the initiation of the electroacupuncture treatment (p = 0.42), but true electroacupuncture significantly reduced the pain felt by patients with RRMS, an effect observed after both three and six months (p = 0.014 and 0.0001, respectively). For the group receiving the sham treatment, there was an improvement after three months, (p = 0.028), but the amelioration was not sustained to the end of the treatment (p = 0.1) (Figure 6).

### Other effects

In addition to the actual results of the study, the participants in the TEA group reported various improvements, such as better sleep and appetite and reduced incontinence and constipation; three participants in this group also reported the disappearance of leg spasms during treatment. Although these effects were not systematically measured, since they were not included in the initial study design, these reports suggest that the positive effects of acupuncture for patients with multiple sclerosis may go beyond the specific aspects of QOL measured. No adverse effects were reported by the patients in either group.

## Discussion

As this study of electroacupuncture on RRMS patients has shown, the use of the technique does indeed improve QOL, which includes the general contentment of these patients, as well as family and social well-being. One of the most incapacitating aspects of the disease is the gradual loss of mobility with the advance of the disease [6]. This study included two measures of this mobility, the EDSS, assessed by a neurologist, and the self-report of mobility subscale of the FAMS. Although the results of the EDSS showed a slightly reduced advance in the results of neurological damage caused by the disease for the patients receiving TEA, the difference between the two groups only approached significance. The results of the mobility subscale of the FAMS, however, showed a truly significant improvement in mobility in the eyes of the patients who had received the TEA. This discrepancy in values is not surprising, since the FAMS score is considerably more subjective, and patient self-impressions are generally not assessed by the EDSS. Furthermore, the latter measures overall performance, and changes on this level take some time to appear.

The degenerative nature of the disease means that actual performance will constantly worsen, and the reduction in mobility of the patients submitted to sham electroacupuncture was noticeable, even in the short six-month period evaluated. The degeneration of those receiving TEA, however, was held in abeyance.

On the other hand, the patient-evaluated QOL is more sensitive to short-term alterations, which would make it possible for the doctor to observe relevant changes in the state of the patient and modify treatment to improve the situation. It is, however, the EDSS which is generally adopted for the evaluation of the mobility of the patient with RRMS. Since the results of this test ignore the quality of the life of the patient, the addition of a test such as FAMS, which does measure it, could be of great benefit to the well-being of a patient.

The life of patients with RRMS tends to be a solitary one for various reasons, not the least of which is the frequent presence of pain, which affect some 29-86% of all patients [26]. A large number of publications point out that patients with chronic pain suffer from reduced social adjustment and an increase in psychiatric morbidity [27-30]. Although pain is rarely considered in the clinical evaluation of RRMS, it does seem important to deal with such a potentially incapacitating aspect of the disease, especially since the results of this study show that it can be efficiently dealt with using electroacupuncture. These results are in agreement with those of previous studies which have demonstrated the analgesic effect of acupuncture, which is known to ameliorate the deregulation of sensory information in the processing of pain [31-36].

The stigmatization of those with RRMS also contributes to the isolation of these patients; this effect is enhanced by many of the symptoms of RRMS, especially depression, which has a strong negative impact on the life of patients [37]. Depression occurs in as many as 60% of patients with multiple sclerosis, although it is independent of the disability inherent in the clinical course of the disease [38]. Not only does depression lead to the isolation of patients from their friends and families, but, when chronic, can even lead to attempts at suicide [6,39]. It can, however, be largely controlled. If the depressive state of a patient can be identified, some of the damage resulting from it can be ameliorated, if not prevented. This study, for example, has shown that electroacupuncture can improve the emotional well being of RRMS patients, with results visible after only three months, although this is not normally assessed in the routine evaluation of RRMS patients.

## Conclusions

A reasonable quality of life is important in stimulating the desire of an RRMS patient to live as full a life as possible. QOL, however, is not a direct reflection of the neurological development of the disease. This paper provides evidence that electroacupuncture can significantly improve various domains of the QOL of MS patients, especially pain. In normal clinical evaluations of the disease, however, emphasis is limited to physical incapacity, and QOL as such is ignored. The regular use of self-evaluation scales such as the FAMS should help identify problems which could be relieved, as well as helping evaluate the efficacy of traditional treatments, since such self-evaluations often reveal more rapid responses to treatment than do the more objective evaluations of trained professionals.

## Competing interests

All authors declare that they have no competing interests.

## Authors’ contributions

JGQC conceived of and organized the study. All authors contributed to its realization, with JGQC, AD, FG, COB, and BPD being largely responsible for the collection of the data, and JGQC, WDS, and LMBS responsible for the organization and analysis of the data. All authors read and approved the final manuscript.

## Pre-publication history

The pre-publication history for this paper can be accessed here:

http://www.biomedcentral.com/1472-6882/12/209/prepub
